# Na^+^,K^+^-ATPase and Cardiotonic Steroids in Models of Dopaminergic System Pathologies

**DOI:** 10.3390/biomedicines11071820

**Published:** 2023-06-25

**Authors:** Alisa A. Markina, Rogneda B. Kazanskaya, Julia A. Timoshina, Vladislav A. Zavialov, Denis A. Abaimov, Anna B. Volnova, Tatiana N. Fedorova, Raul R. Gainetdinov, Alexander V. Lopachev

**Affiliations:** 1 Biological Department, Saint Petersburg State University, Universitetskaya Emb. 7/9, 199034 Saint Petersburg, Russia; shifu1999@yandex.ru (A.A.M.); st059046@student.spbu.ru (R.B.K.); vladislavletsgo@outlook.com (V.A.Z.);; 2 Institute of Translational Biomedicine, Saint Petersburg State University, Universitetskaya Emb. 7/9, 199034 Saint Petersburg, Russia; gainetdinov.raul@gmail.com; 3 Research Center of Neurology, Volokolamskoye Ahosse 80, 125367 Moscow, Russia; timoshina.yu.a@neurology.ru (J.A.T.); abaimov@neurology.ru (D.A.A.); tnf51@bk.ru (T.N.F.); 4 Biological Department, Lomonosov Moscow State University, Leninskiye Gory 1, 119991 Moscow, Russia; 5 Saint Petersburg University Hospital, 199034 Saint Petersburg, Russia

**Keywords:** Na^+^,K^+^-ATPase, cardiotonic steroids, dopamine, bipolar disorder, depression, neurodegeneration

## Abstract

In recent years, enough evidence has accumulated to assert that cardiotonic steroids, Na^+^,K^+^-ATPase ligands, play an integral role in the physiological and pathophysiological processes in the body. However, little is known about the function of these compounds in the central nervous system. Endogenous cardiotonic steroids are involved in the pathogenesis of affective disorders, including depression and bipolar disorder, which are linked to dopaminergic system dysfunction. Animal models have shown that the cardiotonic steroid ouabain induces mania-like behavior through dopamine-dependent intracellular signaling pathways. In addition, mutations in the alpha subunit of Na^+^,K^+^-ATPase lead to the development of neurological pathologies. Evidence from animal models confirms the neurological consequences of mutations in the Na^+^,K^+^-ATPase alpha subunit. This review is dedicated to discussing the role of cardiotonic steroids and Na^+^,K^+^-ATPase in dopaminergic system pathologies—both the evidence supporting their involvement and potential pathways along which they may exert their effects are evaluated. Since there is an association between affective disorders accompanied by functional alterations in the dopaminergic system and neurological disorders such as Parkinson’s disease, we extend our discussion to the role of Na^+^,K^+^-ATPase and cardiotonic steroids in neurodegenerative diseases as well.

## 1. Introduction

It is known that both neurons and glial cells need to constantly restore their resting membrane potentials. Maintenance and restoration of the resting potential is facilitated by Na^+^,K^+^-ATPase (NKA), a cytoplasmic membrane protein complex that exports three Na^+^ ions out of the cell in exchange for two K^+^ ions. This pump action is facilitated by the α subunit, part of a membrane protein complex that also includes the β and γ subunits [1]. In neurons, aside from the ubiquitous α1 isoform, a neuron-specific isoform is present—the α3, while glial cells express the α2 isoform in addition to α1 [2]. Na^+^ export is necessary for neurons to restore the resting potential after the propagation of an action potential, and it facilitates Na^+^-conjugated transport processes [3]. Glial cells use the Na^+^ and K^+^ gradient to transport various compounds across the membrane, including excess neurotransmitters from the synaptic cleft and energy-intensive substrates transported into neurons [4].

A large body of evidence hints at the association of NKA dysfunction with the development of neurodegenerative and neuropsychiatric diseases. For example, mutations in the *ATP1A3* gene cause rapid-onset dystonia parkinsonism (RDP) and alternating hemiplegia of childhood (AHC) [5]. Neurotoxic α-synuclein aggregates, which are a hallmark of Parkinson’s disease, bind to the neuronal α3-subunit of NKA, disrupting its function [6]. Oxidative stress (OS), which can be caused by toxic dopamine metabolites [7], as well as protein kinase C (PKC) activation [8] also cause dysfunction of neuronal NKA. Thus, there is reason to further study the role of NKA dysfunction in pathophysiological processes in the central nervous system (CNS).

In addition to its role in maintaining resting membrane potential, NKA is also involved in a number of intracellular signaling pathways and is a receptor for cardiotonic steroids (CTS), which can induce changes in intracellular signaling when binding to the enzyme. To date, thanks to the use of mass spectrometric analysis, enough data have been accumulated that allow us to consider CTS as endogenous hormone-like compounds in mammals, including humans. Endogenous ouabain was identified in human blood plasma [8,9], and its role in the development of various diseases, including arterial hypertension, was shown [8,9,10]. The presence of marinobufagenin in human blood was identified [11]. Additionally, endogenous CTS were isolated from the bovine adrenal glands [12]. From the bovine hypothalamus, a compound with an integer mass measured by HPLC-mass spectrometry equal to ouabain was isolated by affinity chromatography [13]. Thus, it is assumed that endogenous ouabain can be produced in the brain and adrenal glands of mammals. It has been shown that its amount can increase in response to an increase in tissue NaCl concentration. Increased content of endogenous ouabain in the brain is associated with epilepsy and motor neuron dysfunction [14]. However, there is currently no complete understanding of the physiological role of CTS in the CNS. There is also virtually no knowledge about the pathways of their biosynthesis in the brain and their regulation.

In addition to endogenous CTS, the CNS can also be affected by exogenous factors: the use of the CTS digoxin to treat patients with heart failure can lead to a wide range of neuropsychiatric side effects, such as fatigue, depression, psychosis, and delirium [14,15]. In various experimental models, it was shown that CTS can affect the efficiency of Na^+^ and K^+^-dependent processes by inhibiting NKA [16]. Thus, inhibition of the α3 subunit in neurons leads to the inability to quickly restore the Na^+^ gradient and enable action potential generation [17]. It is also known that ouabain causes increased release of GABA and decreased rate of GABA reuptake [18]. In addition, the NKA in the CNS has a number of functions specific to each isoform that are not directly related to pump activity, including the regulation of other membrane proteins and the activity of intracellular signaling cascades [8]. Via binding to NKA, CTS can influence the work of membrane and cytoplasmic proteins with which they interact [19,20,21]. Experimental data obtained in an amphetamine-induced model of mania in mice indicated the possible involvement of endogenous CTS in the development of bipolar disorder [22]. When entering the bloodstream, endogenous CTS affect the excretory and cardiovascular systems [14]. However, there is currently no complete picture of the involvement of CTS in physiological and pathophysiological processes in the CNS.

In this review, we summarize the data obtained in various models on the role of NKAs and CTS in CNS pathologies related to dopaminergic system dysfunction.

## 2. Neurological Disorders in Animals with NKA Mutations

The α3 subunit of NKA is encoded by the *ATP1A3* gene. To date, four mouse models used to study the in vivo consequences of mutations in the *ATP1A3* gene have been described. The creation of model animals—mice in which the α3 subunit gene promoter (Atp1a3) is used to control the expression of the fluorescent protein ZsGreen1 (*a3NKA-ZsGreen1* mouse model) [23]—made it possible to determine the localization of the α3 subunit in brain tissue. It was shown that the signal intensity was highest in the neuronal bodies located in the stem structures, including the substantia nigra, some nuclei of the thalamus and cerebellum. No fluorescence was detected in astrocytes and brain white matter.

Mutations in the *ATP1A3* gene have an autosomal dominant inheritance pattern. Homozygous mutants die shortly after birth. Therefore, viable and fertile heterozygotes are used to study all four in vivo models. These models display symptoms and endophenotypes similar to those seen in the manic and depressive phases of bipolar disorder, rapid-onset dystonia parkinsonism, epilepsy, alternating hemiplegia of childhood, and CAPOS syndrome to varying degrees (Table 1) [3].

Heterozygous Myshkin mutants (*NKA13AMyk/+; Myk/+*) (1.1 in Table 1) carry a missense mutation with an amino acid substitution at position 810 (I810 N). Such NKA α3 subunits are expressed normally but are not functionally active. Myshkin mutants were originally developed as a preclinical model of epilepsy because heterozygotes exhibited spontaneous seizures [24]. By crossing with seizure-resistant C57BL/6NCr mice, mutants that did not exhibit seizures were obtained [32]. So far, Myk/+ mutants have been shown to be valid models of mania [33]. In behavioral tests, Myk/+ mutants demonstrated hyperactivity, circadian rhythm and sleep disturbances [34], risk-taking tendencies, and increased sensitivity to D-amphetamine [25,35]—these symptoms are seen in patients in the manic stage of bipolar disorder. Additionally, administration of lithium and valproic acid, effective in mania therapy, has been shown to normalize behavior in heterozygous mice. However, it is not known at this time whether an endophenotype of depression is possible in this model in response to stressors. Myk/+ mice were also shown to exhibit a number of disturbances in circadian behavioral rhythms related to the processing of sensory visual information but without disturbances in the function of clock genes [36]. The authors suggested a link between the identified circadian rhythm abnormalities in this mouse model and the sleep disorders observed in parkinsonism. Some reviews on rush-induced dystonia-parkinsonism suggested the use of Myshkin heterozygotes as models of this disease [37]. The 4-week-old Myk/+ displays a different gait than the wild type, unstable with a shorter stride and accompanied by tremor. Tremor and gait problems are symptoms characteristic of parkinsonism. Changes in glucose metabolism and functional brain connectivity have also been shown in mice of this line. However, Myk/+ heterozygotes are not adequate models of RDP and parkinsonism; their endophenotype is more similar to that of alternating hemiplegia of childhood [26].

Heterozygous mutants of Mashlool (α+/D801N; Mashl+/−) (1.2 In Table 1) also carry a missense mutation with an amino acid substitution at position 810. A similar amino acid substitution at the same position is found in AHC patients [38]. Hyperactivity, reduced learning ability, memory problems, tremor, and shorter stride length have been shown for this line of mice compared to wild-type mice. Dystonia, hemiplegia, and hyperexcitability were found in Mashl+/−. In vivo electrophysiology data show that heterozygotes require fewer electrical stimulations for full excitation than wild-type animals; in addition, registration of electrical activity of the amygdala and hippocampus shows that the duration of full excitation of these structures after stimulation is significantly longer in heterozygotes than in wild-type mice. Mashl+/− mutants show spontaneous seizures and have an increased mortality [27]. Mashlool mutant data show that this lineage can serve as an AHC model with some reservations, but it is difficult to judge whether it can be an adequate model for studying bipolar disorder.

Heterozygous mutants with a point mutation in the fourth intron (NKA1A3tm1Ling, NKA1A3+/−, α+/KOI4) (1.3 in Table 1) show an approximately 60% reduction in α3-subunit expression in the hippocampus [28] because of aberrant splicing. At the same time, total NKA activity is reduced by 15% compared to the wild type. Behavioral features of intact (unstressed) heterozygotes are hyperactivity, decreased anxiety, and sensitivity to methamphetamine. No behavioral manifestations of neurological disorders were found in intact heterozygotes [29]. High-performance liquid chromatography showed no change in the levels of serotonin, dopamine, and their metabolites in the striatum in heterozygotes compared to wild-type animals. However, heterozygotes showed increased locomotor activity when presented with methamphetamine, which may be related to disturbances in the dopaminergic system [28]. α+/KOI4 mice exposed to chronic variable stress (CVS) exhibit behaviors similar to those observed in the depressive phase of bipolar disorder: anhedonia, despair-like behavior, weight changes, increased anxiety, and impaired memory and socialization. At the same time, NKA1A3 activity was reduced by 33% compared to the stressed wild type, consistent with the endophenotype of depression [26]. Thus, CVS-treated α+/KOI4 mutants can serve as a model for the depressive phase of bipolar disorder. In males with this mutation, however, no overt symptoms of parkinsonism or dystonia were found before or after stressors. However, for females, chronic stress was shown to induce coordination problems. In addition, rearing in stressed heterozygotes of both sexes was shown to have a negative correlation with levels of dopamine and its metabolites, which was not observed in wild-type mice [29].

Heterozygous Atp1a3tm2Kwk/+ mutants (1.4 in Table 1) have directional deletion of exons 2–6. Hyperactivity in both cell and open field tests was shown for them, but their anxiety level is not significantly different from that of wild-type animals. Heterozygotes have a higher level of coordination and motor balance compared to the wild type. Stressors do not cause dystonia-like symptoms, but microinjections of kainate into the cerebellar vermis induced a similar state. Electrophysiological studies on slides showed a connection of the mutation to the GABAergic system but not to the dopaminergic system [30]. Heterozygotes at 4 weeks of age show a shorter stride length compared to the wild type. Older heterozygotes (6–12 weeks old) do not show gait abnormality in the absence of stressors. However, when exposed to stressors, they begin to take shorter steps when moving, compared to controls. This is very similar to the manifestation of RDP, the symptoms of which in humans can be triggered by stress. It has been suggested that Atp1a3tm2Kwk/+ mutants may be a good model for RDP, although researchers have not reported dystonia or other symptoms of parkinsonism (postural instability, bradykinesia) [31].

For all four genetic models, increased impulsivity, a propensity for risk-taking behavior, and decreased habituation have been shown to varying degrees. All of these behavioral traits are symptoms of mania. The most striking symptoms of a mania-like state are noted in Myshkin mutants. However, there is currently insufficient information about dopamine levels in this line of mice. The depressive phase of bipolar disorder is best reproduced in CVS-exposed NKA1A3tm1Ling mutants. A correlation was found between the activity of stressed mice of this lineage and dopamine levels, but the relationship between dopamine levels and the mania-like state of unstressed heterozygotes carrying this mutation is not well understood.

Gait impairment is one of the symptoms of parkinsonism, including RDP. Gait abnormalities in mice were shown for three of the four models. The Atp1a3tm2Kwk/+ model is the closest to RDP, but it does not demonstrate the full range of classic parkinsonism symptoms. Thus, no genetic model associated with a mutation in the ATP1A3 gene can be called sufficiently reliable to study parkinsonism, at least for the time being. Nevertheless, the manifestation of both manic behavior and motor disorders simultaneously in the models may indicate that mutations in the α3-subunit of NKA can phenotypically manifest these two pathologies. Further research is needed to understand the mechanisms of the relationship between these pathologies.

Mutations that disrupt the α2-subunit of NKA, which is expressed in the brain in glial cells, can also lead to the development of various neurological and neuropsychiatric disorders. Variants in the ATP1A2 gene, which encodes the α2-subunit of NKA, are associated with familial hemiplegic migraine. For example, patients with the G301R mutation are affected by a complex syndrome characterized by migraine comorbidity with epilepsy, motor symptoms, and depression or obsessive–compulsive disorder [39,40]. This mutation was successfully replicated in mice, which displayed impaired glutamate uptake and altered inflammatory cytokine signaling [39,40].

## 3. Using Cardiotonic Steroids to Model Dopaminergic System Dysfunction

In addition to using animal lines with mutations in the NKA genes, studies of the effect of NKA dysfunction on the dopaminergic system have been conducted using intracerebroventricular (ICV) administration of ouabain to laboratory animals. The first indication of CTS involvement in affective disorder pathogenesis was seen in patients with heart failure, who developed mania-like symptoms in response to treatment with digoxin [15]. After this discovery, a series of attempts was made to model BD using ouabain, which, like digoxin, is a cardenolide. The first report of mania-like behavior after ICV injection of ouabain in rats was published in 1995 [41]. Since then, two approaches to modeling BD using ICV ouabain injection have emerged.

The first category of models includes administration of highly concentrated ouabain during a stereotaxic operation into the lateral ventricle of an anesthetized animal, with a subsequent behavioral evaluation 7–10 days post injection. This approach showed that a single ICV injection of 5 µL of 1 mM ouabain causes increased locomotion and grooming frequency in rats 11 days post injection, accompanied by decreased phosphorylation of PI3K, Akt, and GSK3β, and unchanged ERK1/2 phosphorylation. Seven days post ouabain injection, oxidative changes were observed in brain tissue [42]. Both the manic and depressive phases of BD were present in this model [43]. Chronic administration of valproate, lithium, or AR-A014418 (an inhibitor of GSK3β) prevented all of the above [44,45]. Haloperidol, a D2 receptor antagonist, also prevented ouabain-induced hyperlocomotion in rats in concentrations that decrease locomotor activity in intact animals [46]. It was also shown that the mania-like behavior observed in this model was accompanied by PKC activation [47]. Fourteen days post ouabain injection, animals displayed locomotor depression and impaired memory. Levels of pro-BDNF and BDNF in the frontal cortex were found to be decreased on the 7th day post injection, while its receptor (TRKB) and CREB decreased on the 7th and 14th day post injection [48]. On the 14th day post ouabain administration, the observed depressive symptoms were accompanied by increased levels of interleukin IL-1β, IL-6, IL-10, TNF-α, and CINC-1 in the frontal cortex and hippocampus [49], which may indicate the development of neuroinflammatory processes. A similar model was developed in mice, where anesthetized animals were given ICV injections of 0.625 pmol ouabain. After 8 days, the animals developed signs of mania-like behavior accompanied by c-fos activation. Administration of lithium chloride and haloperidol also neutralized the effects of ouabain in this model [50].

The second category includes models where ouabain is administered to unanesthetized animals using a surgically implanted cannula, and the effects are observed immediately post injection and/or several days later. Thus, an increase in motor activity within 30 min after ICV injection of ouabain (5 µL 0.5–1 mM) was described. At the same time in the striatum, there was an increase in the phosphorylation of ERK1/2 and tyrosine hydroxylase (TH). Administration of the MEK1/2 inhibitor (ERK1/2 MAP kinase kinase) U0126 leveled the effect of ouabain on the motor activity of the animals [51]. In another study of this model, animals injected with ouabain were shown to have increased phosphorylation of Akt, GSK3β, FOXO1, and eNOS amid increased motor activity 1–8 h after ICV injection [52]. At the same time, chronic administration of lithium chloride (for 7 days before ICV injection of 5 μL of 1 mM ouabain) was shown to prevent an increase in motor activity in rats [53]. The mechanism of the effect of ouabain in this model was attributed to mTOR activation mediated by Akt and ERK1/2 activation, with a subsequent effect on the expression of a number of proteins [54]. In a recently published paper, we described a model of ouabain-induced mania in mice. ICV injection of 0.5 μL of 50 μM ouabain into the lateral ventricles of the brain caused an increase in motor activity and stereotypic movements, as well as a decrease in anxiety in the animals within 1 h after the injection. At the same time, ouabain was shown to cause a decrease in the rate of dopamine reuptake. Inhibitor analysis with haloperidol showed that the effects of ouabain were mediated by the activation of D2 dopamine receptors and were associated with Akt activation, GSK3β deactivation, and ERK1/2 kinase activation, but not with neurodegenerative changes, which were not detected in animals 24 h after ouabain administration [55].

It would seem that the models described above are associated with the administration of CTS in doses that significantly exceed physiological ones. However, there is evidence that in other models of mania in laboratory animals endogenous CTS play a role in the development of the pathophysiological process. Thus, the administration of anti-ouabain antibodies, which reduced amphetamine-induced hyperactivity, protected against OS in the brain [56]. Moreover, administration of the ouabain antagonist rostafuroxin ameliorated behavioral and brain biochemical changes in the dextromethorphan-induced mania model [57].

Based on the data obtained in these models, we can conclude that NKA dysfunction induced by the administration of both exogenous CTS and endogenous CTS may be associated with dopaminergic system dysfunction, causing symptoms of neuropsychiatric diseases. However, these studies have not shown neurological abnormalities and degeneration of dopaminergic neurons. The only study on modeling Parkinson’s disease (PD) with CTS was conducted on Danio Rerio, where the CTS neriifolin [58] was used as a parkinsonism inducer.

## 4. Evidence for NKA Dysfunction in Experimental PD Models

At the same time, there is ample evidence of NKA impairment in classical models of parkinsonism. The most widespread method for modeling PD in laboratory rodents utilizes mitochondrial toxins, such as 1-methyl-4-phenyl-1,2,3,6-tetrahydropyridine (MPTP), rotenone, and 6-hydroxydopamine (6-OHDA). Mitochondrial dysfunction accompanied by OS are both involved in DAergic neuron degeneration. Because of the high energy demands of NKA—its activity can account for over 50% of neuronal ATP consumption [59]—it is extremely sensitive to mitochondrial dysfunction. Because of its large number of modifiable sites, it is also sensitive to reactive oxygen species (ROS) [60].

In mouse MPTP-induced parkinsonism, an approximately 40% decrease in total NKA activity in the striatum occurs, accompanied by a 60% decrease in dopamine levels [61]. A 60% decrease in NKA activity was also observed in conditions of 1-methyl-4-phenylpyridinium (MPP^+^)-induced OS in NGF-differentiated pheochromocytoma of the rat adrenal medulla (PC12) cells [62]. NKA activity also decreases in rotenone-induced parkinsonism models—total brain activity by approximately 25–40% [63,64] and the midbrain and striatum by 22% and 28%, respectively [65]. One of the first effects of exposure to rotenone is intracellular accumulation of sodium, which causes early hyperpolarization and a build-up of intracellular calcium following depolarization [66]. This coincides with changes in ion traffic that occur post NKA inhibition with ouabain [67], suggesting that these changes in rotenone-induced parkinsonism are a direct consequence of NKA dysfunction due to impaired ATP synthesis. Exposure to 6-OHDA, which inhibits all four mitochondrial electron transport chain complexes, causes a 28–43% decrease in NKA activity accompanied by a significant decrease in DA and its metabolites [68,69].

## 5. Mechanisms and Positive Feedback Loops: NKA and DAergic System Dysfunction

Factors affecting NKA function can be divided into two broad categories, the first including non-specific factors such as ATP concentration; Na^+^, K^+^, Mg^2+^ concentrations; OS; phosphorylation by various intracellular kinases; misfolded protein aggregates (α-synuclein, β-amyloid, superoxide dismutase); and various modifications (including glutathionylation), and the second including specific ligands—CTS. Non-specific factors can be divided into factors that increase NKA activity and those that decrease it. For example, phosphorylation by PKC, OS, low ATP, and interaction with misfolded protein aggregates cause a decrease in NKA activity. Factors such as glutathionylation and increased intracellular Na^+^ or extracellular K^+^ concentrations cause an increase in NKA activity. In turn, CTS exert different effects depending on the CTS and the concentration—concentrations below 10 nM can induce an increase in NKA activity, while concentrations exceeding 10 nM inhibit it [70,71]. Via binding to E2P conformation of NKA [72], different CTS can lead to the activation of various intracellular signaling pathways, which was discussed previously by other authors [73,74]. As such, in this review, we will focus specifically on the effects that altered NKA function may have on dopamine signaling and metabolism.

As one of the main functions of NKA is the maintenance of the electrochemical gradient, alterations in its function inevitably affect Ca^2+^ signaling. Since the pacemaking activity of dopaminergic neurons, specifically those in the substantia nigra, is dependent on intracellular Ca^2+^ oscillations and continuous Ca^2+^ influx [75,76], dysregulation of Ca^2+^ oscillations via NKA inhibition may synergize with exposure to other risk factors, causing mitochondrial damage via oxidative stress [75,77]. Indeed, it was shown previously that Ca^2+^ influx in dopaminergic neurons is a feed-forward mechanism that stimulates mitochondrial oxidative phosphorylation [78], thus increasing metabolic load. Considering that dopaminergic neurons experience high basal metabolic load compared to other neuron types, NKA dysfunction-induced Ca^2+^ homeostasis alterations could contribute to dopaminergic neuron degeneration.

**Figure 1:**
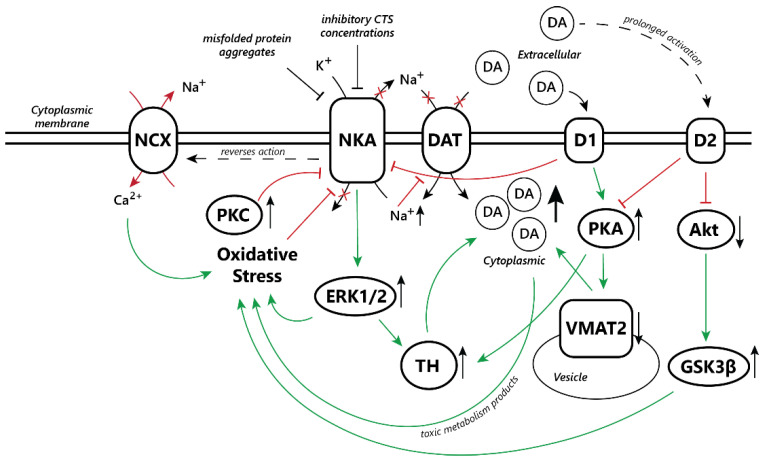
Potential consequences that NKA dysfunction, whether from high CTS or other pathological conditions, may have on dopaminergic signaling. Green arrows represent downstream activation, while red arrows represent inhibitory processes. Short black arrows to the right of a given element denote increased activation or increase in concentration (pointing up), or decreased activation or decrease in concentration (pointing down).

Ca^2+^ and NKA signaling in neurons was extensively discussed in a recent review by Kinoshita et al. [67], and as such we will not go into detail on the subject. In brief, CTS are known to influence Ca^2+^ homeostasis in different ways depending on the CTS and concentration. In low, nanomolar concentrations, CTS can cause Ca^2+^ oscillations in neurons, mediated by the direct protein interaction of NKA with the inositol 1,4,5-trisphosphate receptor (IP3R). Low concentration ouabain-induced Ca^2+^ oscillations were shown to promote dendritic growth in an embryonic culture of primary cortical neurons [79] and improve long-term spatial reference memory in rats when administered into the hippocampus [80]. As such, at low concentrations ouabain is considered to have a neuroprotective effect on some neurons through its activation of CREB, the Wnt/β-catenin pathway, and NF-κB [81]. In subnanomolar concentrations, ouabain also protects against NMDA-induced cytotoxicity via direct protein-to-protein interactions between NKA and the Na^+^/Ca^2+^ exchanger [20]. In concentrations that inhibit NKA, CTS binding slows down or reverses the action of the Na^+^/Ca^2+^ exchanger, which co-localizes with NKA, thus increasing local cytoplasmic Ca^2+^ and leading to glutamate-mediated excitotoxicity [82] (Figure 1).

**Figure 2:**
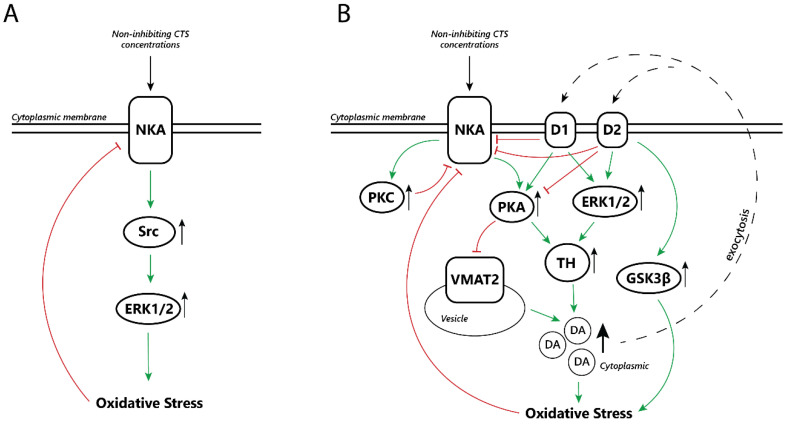
OS caused by non-inhibitory concentrations of CTS, mediated via the Src-ERK1/2 pathway (**A**); possible consequences of chronic elevation of endogenous (non-inhibiting NKA) CTS concentrations in dopaminergic system neurons (**B**). Green arrows represent downstream activation, while red arrows represent inhibitory processes. Short black arrows to the right of a given element denote increased activation or increase in concentration (pointing up), or decreased activation or decrease in concentration (pointing down).

On the basis of the available data, it is possible to suggest several hypotheses of how changes in NKA functioning, due to both fluctuating CTS levels and other factors, can lead to dopaminergic neuron death. In the above-described models, CTS cause an increase in dopamine receptor activation. This may be a consequence of impaired dopamine reuptake, increased dopamine release, or increased dopamine synthesis, as has been demonstrated in various studies [51,55]. For different tissue types and cell cultures, it was shown that CTS in non-inhibitory concentrations can cause OS via activation of the Src-ERK1/2 signaling pathway [83,84] (Figure 2A). In turn, we propose a pathway that can lead to non-inhibitory CTS concentrations causing OS specifically in dopaminergic neurons (Figure 2B).

Inhibition of NKA activity by 40–50 µM ouabain in mouse striatum slices was shown to induce a decrease in the rate of DA reuptake by the dopamine active transporter (DAT) and an increase in its duration in the synaptic cleft. Reduced DAT activity normally causes activation of D2 dopamine autoreceptors on the presynaptic membrane, increasing the rate of dopamine transport from the cytoplasm to vesicles via the vesicular monoamine transporter-2 (VMAT2) [85,86,87]. Long-term dysfunction of DAT leads to increased duration of DA circulation in the synaptic cleft [55]. In DAT gene knockout mice, it was shown that DAT dysfunction leads to a decrease in presynaptic D2 autoreceptors [88]. Thus, long-term DAT dysfunction can lead to both an increase in DA synthesis [89] and a decrease in its uptake into vesicles by VMAT2. VMAT2 dysfunction is known to be associated with the development of PD owing to the accumulation of toxic products of DA metabolism [90]. Moreover, people with DAT dysfunction develop juvenile parkinsonism (with complete loss of function in the first months of life, with partial loss of function in adolescence), whereas partial loss of function leads to the development of bipolar disorder [91] (Figure 1).

Previously it was shown that ICV ouabain administration causes an increase in TH phosphorylation via ERK1/2 activation, indicating that DA synthesis increases as well [51]. ERK1/2 activation post ouabain injection was demonstrated several times, both in vivo in rodents [51,55,92] and in vitro on neuron cultures [93]. Although it is known that ouabain can activate PKA and PKC in rat cortex neuron cultures [94], to our knowledge there have been no studies showing that ouabain-induced TH activation is mediated by these kinases. In various cell cultures, however, it has been shown that PKA activates TH via Ser40 phosphorylation [95,96]. Furthermore, PKA activation leads to an increase in TH expression [97]. It was also shown that phorscoline-induced PKA activation causes an increase in DA release in rat striatum slices [98] and increased D2R expression in the striatum post ICV administration in rats [99]. PKA activation also causes an increase in DAT activity in rat striatum-derived synaptosomes [100]. On the other hand, PKA inhibition in PC12 cell culture causes an increase in VMAT2 amounts in “synaptic” vesicles [101].

It is likely that post a single injection of ouabain, TH activity eventually returns to normal. However, if endogenous CTS levels in the brain remain elevated chronically, similar to blood plasma levels of CTS in hypertension [14], it is possible that the observed neuronal TH hyperactivity is sustained chronically as well. It is known that TH hyperactivation in neurons leads to the accumulation of toxic dopamine oxidation products, OS, and eventually neuron death [102]. In addition, it was shown that hyperstimulation of dopamine receptors can lead to neuronal death [103]. Prolonged D2R activation is known to trigger a β-arrestin-dependent signaling pathway, leading to increased GSK3β activity [104,105]. Pathological GSK3β activity is known to be associated with DA neuronal degeneration and PD [106]. Activation of GSK3β also causes NURR1 degradation [107], which is vital to VMAT2 expression [108]. One of the mechanisms responsible for neuronal death during GSK3β hyperactivation is an increase in NR2B-containing NMDAR activity followed by Ca^2+^ overload [109]. Thus, we can assume that NKA dysfunction is associated with OS and other stressors (including products of DA metabolism [7]).

As mentioned above, ERK1/2 activation and increased TH phosphorylation in the striatum is characteristic of CTS-induced mania-like behavior models [51]. It is known that activation of ERK1/2 in primary culture neurons can be induced by various CTS and is associated with the neurotoxic effect of ouabain [91,110]. In the described models, activation of ERK1/2 also occurs upon administration of ouabain. ERK1/2 is known to play an ambiguous role in the pathogenesis of PD. ERK1/2 activation is necessary for the implementation of protective mechanisms in neurons when exposed to stress factors that lead to the initiation of neurodegeneration. PI3K/Akt and ERK1/2 signaling pathways are known to be involved in protecting dopaminergic neurons from MPTP/MPP^+^-induced neurotoxicity [111]. Previously, it was shown that ERK1/2 is involved in neuronal antioxidant defense and translocating to the nucleus via binding to the DJ-1 protein [112]. Increased amounts of p-ERK1/2 were found in the mitochondria of degenerating neurons from PD patients and patients with dementia with Levi’s corpuscles [113]. Other studies supported the idea that ERK1/2 inhibition causes activation of both apoptotic and necrotic pathways, leading to neuronal death [114]. On the other hand, activation of ERK1/2 and JNK is known to be associated with L-DOPA-induced neurotoxicity to dopaminergic neurons in a cellular model of PD [115]. In PD models, ERK1/2 activation mediates the occurrence of OS in pro-inflammatory factor-activated microglia. ERK1/2 is also involved in the development of L-DOPA-induced dyskinesia by affecting synaptic plasticity in the striatum [116,117]. Using the CG4 oligodendroglial cell line, it was shown that H_2_O_2_-induced cell death is prevented by the ERK1/2 pathway inhibitor PD98059 [118]. PD98059 can also prevent neuronal degeneration caused by nitric oxide released by glial cells through ERK1/2 activation [119]. The use of another inhibitor, U0126, also demonstrated that dopamine-induced striatal neuronal death is associated with ERK1/2 activation [120].

Dopamine binding to dopamine receptors can decrease NKA activity through PKC and PKA activation [70]. Dopamine binding to the D1 dopamine receptor in striatum neurons leads to a decrease in NKA activity. Binding of dopamine to the D2 dopamine receptor induces sodium channels to open, causing a spike in intracellular Na+ concentration and activating NKA [121]. Using co-immunoprecipitation and mass spectrometry, it was shown that D1 and D2 dopamine receptors form a protein complex with NKA. Transfection of the D1 or D2 dopamine receptor into HEK293T cells without dopamine addition resulted in a marked decrease in α1-containing NKA activity but had no effect on its amount [122]. Furthermore, as mentioned earlier, OS and PKC activation also cause a decrease in NKA activity, closing the positive feedback loop.

Thus, there are many ways in which chronic NKA dysfunction due to a chronic increase in endogenous CTS in the brain or due to other factors affecting NKA may lead to the degeneration of dopaminergic neurons.

## 6. Conclusions

Although there is currently no clear picture of the role of CTS and NKA abnormalities in the development of neurodegenerative diseases of the dopaminergic system, there is an understanding of their role in the development of affective disorders associated with functional dopaminergic pathologies. That being said, there is a significant amount of evidence suggesting that CTS and NKA abnormalities may be key players in the development of neurodegenerative disorders of the DA system such as PD. Further study of changes in both NKA functioning and the amount of endogenous CTS in neurodegenerative disorders of the DA system, and mechanisms of CTS influence on the dopaminergic system in various models at the physiological, neurochemical, and biochemical levels could open up potential new pharmacological targets and biomarkers for both PD and affective disorders.

## Figures and Tables

**Table 1:** *ATP1A3* genetically modified models.

Model	Symptoms of Affective Disorders	Symptoms of Neurological Disorders	In Vivo Electrophysiology Data	Changes in Dopamine Levels	References
1.1 *Myk/+*–	Mania: Hyperactivity Sleep disturbances Dysregulated circadian rhythm Tendency to engage in high-risk behavior Increased sensitivity to amphetamine Decreased anxiety High impulsivity Lower spatial memory	Tremor Impaired gait	-	-	[24,25,26]
1.2 *Mashl+/−*	Mania: Hyperactivity Increased excitability Decreased anxiety High impulsivity Lower spatial memory	Tremor Impaired gait	High excitability, prolonged arousal after a threshold stimulus	-	[27]
1.3 *NKA1A3tm1Ling*	Mania: Hyperactivity Increased sensitivity to amphetamine Decreased anxiety Impulsivity Low habituation Depression: Anhedonia Despair-like behavior Increased anxiety Impaired learning and memory Decreased socialization	-	-	Mania: Not different from wild type Depression: Negative correlation with vertical activity	[25,28,29]
1.4 *Atp1a3tm2Kwk/+*	Mania: Hyperactivity Impulsivity Lower spatial memory	Impaired gait Symptoms similar to RDP	-	-	[30] [31]

## Data Availability

Data sharing not applicable.
